# Modeling realistic extracellular spiking activity in populations of neurons for the purpose of evaluating automatic spike-sorting algorithms

**DOI:** 10.1186/1471-2202-13-S1-P143

**Published:** 2012-07-16

**Authors:** Espen Hagen, Torbjørn Bækø Ness, Amir Khosrowshahi, Felix Franke, Gaute T Einevoll

**Affiliations:** 1 Department of Mathematical Sciences and Technology, Norwegian University of Life Sciences, 1432 Ås, Norway; 2 Redwood Center for Theoretical Neuroscience, University of California, Berkeley, CA 94720-3198, USA; 3 Bio Engineering Laboratory, ETH Zürich, CH-4058 Basel, Switzerland

## 

The emergence of silicon-based extracellular recording devices with large numbers of electrode contacts, such as multi-shank laminar electrodes or high-density multi-electrode arrays (MEA) now makes it possible to potentially record spiking activity from thousands of neurons simultaneously. With the concomitant increase in amounts and complexity of data, manual spike sorting is no longer an option, and there is a dire need for automatic spike-sorting methods. These methods must be able to correctly and efficiently resolve spikes of individual neurons from the recorded mixture, as discussed in a recent review [1].

**Figure 1:**
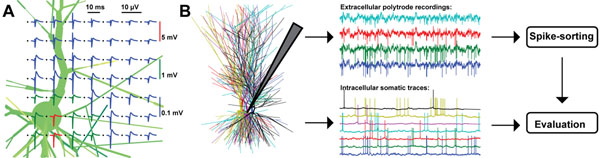
**A.** Position-dependent extracellular spike shapes in vicinity of a cat L5 pyramidal neuron, obtained using LFPy. **B.** Schematic of the evaluation of spike-sorting methods against ground-truth test data for a population of hippocampal pyramidal neurons.

For assessing the sorting quality, spike sorting methods should ideally be evaluated against test data with known ground truth, i.e., data for which the underlying spiking activity of each neuron in the recorded population is known. Unfortunately, such data sets cannot easily be acquired from experiments. One remedy is realistic, model-based simulations of extracellular recordings. Position-dependent spike shapes (Figure 1A) can straightforwardly be modeled in a biophysically realistic way by means of a recently released modeling tool, LFPy (compneuro.umb.no/LFPy), a Python implementation of a forward-modeling scheme [2] for extracellular potentials based on calculations of transmembrane currents in NEURON [3].

Test data for arbitrary electrode geometries, model neurons, noise levels and synchronicity levels can be produced at wish. Here we present example results relevant for tetrode and polytrode recordings in cortex and hippocampus. We also present test data for situations where prominent electrical boundary-condition effects significantly modify the recorded potentials, and FEM modeling must be used to solve the electrostatic problem. This is of particular relevance for cell cultures or retinal slices placed on high density MEAs.

An algorithm evaluation website has been set up on http://www.g-node.org/spike to facilitate use of such benchmark data to evaluate the performance of spike sorting algorithms, as outlined in Figure 1B.
